# Effects of Nitrogen Forms on Soil Enzyme Activities in a Saline‐Alkaline Grassland

**DOI:** 10.1002/ece3.70501

**Published:** 2024-10-30

**Authors:** Jiangqi Wu, Haiyan Wang, Guang Li, Fujiang Hou, Guorong Xu

**Affiliations:** ^1^ State Key Laboratory of Aridland Crop Science Gansu Agricultural University Lanzhou China; ^2^ College of Forestry Gansu Agricultural University Lanzhou China; ^3^ College of Urban Environment Lanzhou City University Lanzhou China; ^4^ State Key Laboratory of Grassland Agro‐Ecosystems, Key Laboratory of Grassland Livestock, Industry Innovation, Ministry of Agriculture, College of Pastoral Agriculture Science and Technology Lanzhou University Lanzhou China

**Keywords:** nitrogen forms, saline‐alkaline grassland, soil enzyme activities

## Abstract

Global climate change and agricultural practices have increased atmospheric nitrogen (N) deposition, significantly affecting the nitrogen cycling process in grasslands. The impact of different N forms on key soil enzyme activities involved in N nitrification, particularly in the saline‐alkali grasslands of the Hexi Corridor, using natural grassland as a control (CK) and adding three N treatments: inorganic N (IN), organic N (ON) and a mixed N treatment (MN, with a 4:6 ratio of organic to inorganic N). Our study assessed the effects of these N forms on soil properties and enzyme activities crucial for N cycling. The findings indicate that different N forms significantly enhance soil mineral N content, with ON treatment leading to the highest increases in nitrate and ammonium content 92.44% and 35.6%, respectively, compared to CK. Both IN and ON treatments significantly boosted soil nitrate reductase and urease activities (*p* < 0.05), while MN treatment decreased nitrate reductase activity, with ON treatment showing the greatest sensitivity to enzyme activity changes. Soil pH slightly increased with N addition, but soil nitrite reductase activity remained relatively unchanged (0.372–0.385 mg g^−1^). Correlation analysis revealed that soil mineral N content and pH are key regulators of enzyme activities in saline‐alkaline grasslands. These results suggest that different N forms should be considered in nutrient cycling models, with organic N addition potentially enhancing soil N conversion and mitigating nutrient limitations in grassland ecosystems.

## Graphical abstract

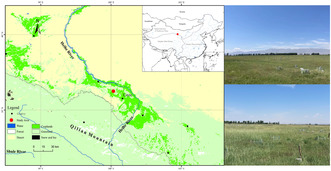

## 1 Introduction

Soil enzymes, as biocatalysts, are vital to carbon (C) and nitrogen (N) cycles, influencing material cycling and energy conversion in soil ecosystems (Tahir et al. 2023). Over recent decades, atmospheric N deposition has increased due to intensive agriculture and fossil fuel combustion (Wang et al. 2024). In China, the annual average N deposition has risen from 13.2 kg ha^−1^y^−1^ to 21.1 kg ha^−1^y^−1^, with a growth rate of 0.41 kg ha^−1^ annually (Liu et al. 2013). N‐containing compounds from human activities, such as grazing and agriculture, have altered N deposition patterns in terrestrial ecosystems, affecting soil enzyme activities and N cycling (Chen et al. 2019). Organic N comprises about 28% of the total N deposition in China, with regional variation between 7% and 67% (Dong et al. 2020). Most studies simulate atmospheric N deposition by adding a single N form, N deposition (Tian and Niu 2015). Thus, examining the effects of various N forms is crucial for understanding their impact on soil enzyme activities and nutrient cycling in saline‐alkaline grasslands.

Grasslands are China's largest terrestrial ecosystem, covering 41.7% of the land area and are critical for ecological sustainability and food security (Michalk et al. 2019; Liu et al. 2023). N is typically the primary limiting factor for productivity in grassland ecosystems (Chen et al. 2019). Diverse N deposition forms, whether natural or anthropogenic, can alter soil enzyme activities and nutrient cycling, influencing ecosystem C and N cycles and providing feedback on global climate change (Reich et al. 2018; Piao et al. 2019; Vikram, Chaudhary, and Rao 2024). Urease, nitrate reductase and nitrite reductase are key enzymes in the N cycle (Pu et al. 2019; Timilsina et al. 2024) involved in essential processes like N fixation, ammonification, nitrification and denitrification, which are critical for global nutrient cycling. Urease catalyzes urea hydrolysis into ammonia and carbon dioxide, reflecting the soil's capacity to supply inorganic N (Solangi et al. 2024), while the activity of soil nitrate reductase and nitrite reductase reflects the strength of soil denitrification ability (Wan et al. 2023). In grasslands, atmospheric N deposition contributes about 40% of organic N (Zhang et al. 2012), supplemented by sources like animal feces (Cai and Akiyama 2016). Different N compounds affect soil enzyme activities by modifying substrates, soil C fractions and microbial community structures (Zhao et al. 2022; Jia et al. 2019; Wang et al. 2019). These changes, in turn, influence soil fertility transformation N utilization.

With the advancement of the “One Belt, One Road” initiative, the Hexi Corridor has become a vital ecological barrier in the northwest of China, providing essential resources for local communities (Sahab et al. 2021). However, natural and anthropogenic factors have led to significant salinization in this region, with 79% of China's saline‐alkaline soils located in the Hexi Corridor (Sahab et al. 2021; Zhao, Zhong, and Pan 2018). Soil salinization, a major constraint on grassland productivity and pasture quality, affects root uptake of water and nutrients, thus impacting grassland productivity (Liu et al. 2020). Soil salinity and pH, as important factors and determinants of enzyme activity, influence nutrient transformation processes and ecosystem C and N cycles (Brown, Rhymes, and Jones 2022). Research on saline‐alkaline grasslands in the Hexi Corridor has largely focused on management practices (Cheng, Chen, and Zhang 2018; Shakoor et al. 2022) and vegetation types (Yang et al. 2022), with limited understanding of how different N forms affect soil properties and enzyme activities.

This study focuses on the central Hexi corridor, where we established experimental plots with four N treatments to investigate soil factors and enzyme activity responses. Our objectives were (1) to study the effects of different N forms on soil N‐cycle enzyme activities in saline‐alkaline grasslands; (2) to analyzing the relationship between N‐cycle enzyme activity and soil water content, pH, and inorganic N components. Our aim is to provide a theoretical basis for predicting ecological changes in saline‐alkaline grasslands due to human activities. We hypothesized that (1) N addition in N‐limited grassland ecosystems increases soil nitrate and ammonium N content, with varying effects depending on the N form and (2) inorganic N or organic N addition enhances soil urease and nitrate reductase activities, while mixed N fertilizers significantly reduce these activities.

## 2 Materials and Methods

### 2.1 Experimental Location

**FIGURE 1:**
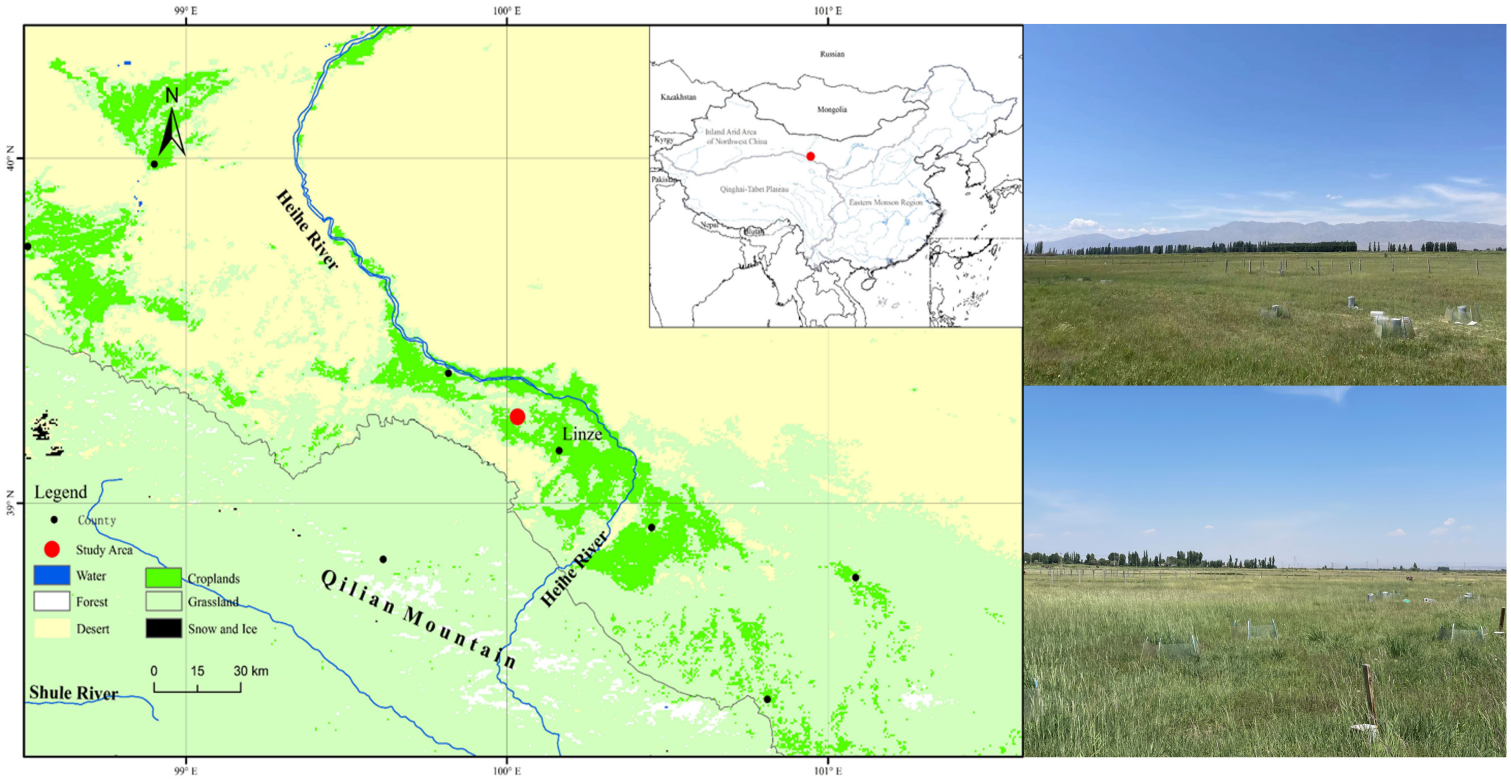
Location of sampling sites in saline‐alkaline grasslands.

This study was conducted at the Lanzhou University Grassland Agricultural Experiment Station, located in Linze County, Gansu province (100°02′ E, 39°15′ N) (Figure 1). The study area is situated at an average elevation of 1390 m and is characterized by a temperate continental climate with dry springs and cold, windy winters. The average annual temperature is 7.16°C, with an annual precipitation of 121.5 mm, mostly occurring in summer, fall and a potential annual evaporation rate of approximately 2337.6 mm. The area consists of saline‐alkaline grassland and desertified grasslands, with highly salinized soil, poor permeability, low porosity and sparse vegetation. The dominant vegetation types include *Poa annua*, *Leymus secalinus*, *Phragmites australis* and *Kalidium foliatum*.

### 2.2 Experimental Design

In February 2022, three replicated plant transects were established in the saline‐alkaline grassland of Linze, selecting areas with flat terrain, uniform vegetation types and consistent coverage. Different forms of N fertilizer were applied to each plant transect using a complete block design. Each plot measured 5 m × 5 m, with 40 m between transects and 5 m between plots within a group, resulting in a total of 12 plots. Natural grasslands were used as a control treatment (CK). Inorganic N fertilizer (IN, using NH_4_NO_3_), organic N fertilizer (ON, urea: glycine = 1:1) and mixed N fertilizer (MN, ON: IN = 4:6, reflecting the highest proportion of atmospheric N deposition in temperate grasslands) were applied at a rate of 15 kg N hm^−2^ y^−1^, consistent with current total N deposition across Chinese grasslands (Dong et al. 2020). At the end of May 2022, the fertilizers were weighed, mixed evenly with 5 L of groundwater and sprayed to prevent surface runoff in the sample plots. The CK plot was treated with 5 L of groundwater only.

### 2.3 Sample Collection and Determination of Parameters

Soil samples were collected in mid‐June 2022, using a soil auger, following the “S” five‐point method at depths of 0–10, 10–20 and 20–40 cm. Samples from the same soil layer within each plot were combined, sieved through a 2‐mm sieve, with plant residual roots, stones and debris removed, then packed into sealed bags and kept on ice. One sub‐sample was stored at 4°C for determining (Yang et al. 2021) soil physicochemical indices including pH, ammonium nitrogen (NH_4_^+^‐N) and nitrate nitrogen (NO_3_^−^‐N). Another sub‐sample was air‐dried to assess (Liu et al. 2021) soil nitrogen cycling enzyme activity (urease, nitrate reductase, nitrite reductase). All soil indicators were evaluated in triplicate, with the average value used for data analysis.

Soil water content (SWC) was determined using a drying method. Soil NH^+^_4_‐N and NO^−^_3_‐N were quantified using the MgO‐Deichmann alloy distillation method, according to Wu et al. (2021). Soil pH was measured with a pH meter after leaching at a water‐soil ratio of 2.5:1. Urease activity was characterized spectrophotometrically, following methods by Ge et al. (2010) and Yin et al. (2014). Soil nitrate and nitrite reductase activities were determined using the benzenesulfonic acid‐acetic acid‐α naphthylamine colorimetric method as described by Zhao et al. (2022). For urease activity, 6 mL of urea solution and 12 mL of citrate buffer were added to 3 g of soil and incubated at 37°C for 24 h. Post‐incubation, 4 mL of sodium phenolate solution and 3 mL of NaClO solution were added, the suspension was shaken for 30 min and absorbance was measured at 578 nm. For nitrate reductase activity, 1 mL of 0.8 M 2, 4‐dinitrophenol solution, 1 mL of 0.1 M potassium nitrate solution, 1 mL of 0.1 M glucose solution and 5 mL of distilled water were added to 1 g of soil sample and incubated at 30°C for 24 h, followed by adding 1 mL of alumina‐potassium alum saturated solution and shaking for 30 min. A sample of 1 mL of filtrate was then combined with 4 mL of color reagent (α‐naphthylamine‐sulfanilic acid) and incubated for 15 min before measuring absorbance at 520 nm. For nitrite reductase activity, 2 mL of sodium nitrite solution (0.25 M) and 5 mL of distilled water were added to 1 g of soil and incubated at 30°C for 24 h. The subsequent measurement method is consistent with nitrate reductase activity.

### 2.4 Statistical Analysis

One‐way ANOVA (SPSS 20.0) was employed to analyze differences in SWC, pH, NH_4_^+^‐N, NO_3_^−^‐N and soil enzyme activity across different treatments and soil depths, with significance set at *p <* 0.05. Pearson correlation analysis was conducted to examine relationships between soil enzyme activities and selected soil factors, with plots created using Origin 2021.

## 3 Results

### 3.1 Effects of N Treatments on SWC and pH

SWC and pH showed significant variation across treatments (IN, ON and MN) and the control (CK) (Table 1). Compared to CK, SWC under IN, ON and MN treatments gradually decreased, the SWC following a trend of an initial decrease followed by an increase with soil depth. Soil pH in the 10–40 cm layer under MN treatment was significantly higher than the other treatments (*p* < 0.05), showing a gradual increase with soil depth, while pH in other treatments decreased and then increased with depth. Repeated analysis of variance demonstrated that N addition and soil depth significantly interaction influenced the soil SWC and pH (Table 2).

**TABLE 1:** SWC and pH under different N treatments at selected soil depths (means ± standard errors).

	Treatments	0–10 cm	10–20 cm	20–40 cm	0–40 cm
SWC	CK	0.417 ± 0.010 Aa	0.374 ± 0.001 Ab	0.429 ± 0.001 Ba	0.407 ± 0.003 A
	IN	0.396 ± 0.003 ABb	0.369 ± 0.002 Ac	0.439 ± 0.001 Aa	0.401 ± 0.002 A
	ON	0.393 ± 0.001 Ba	0.357 ± 0.001 Bb	0.395 ± 0.000 Ca	0.382 ± 0.001 B
	MN	0.403 ± 0.002 ABa	0.352 ± 0.001 Cb	0.353 ± 0.001 Cb	0.383 ± 0.001 B
pH	CK	7.647 ± 0.066 Aab	7.572 ± 0.030 Cb	7.716 ± 0.011 Ca	7.645 ± 0.033 B
	IN	7.718 ± 0.053 Aa	7.673 ± 0.032 Ba	7.743 ± 0.003 Ba	7.711 ± 0.028 B
	ON	7.704 ± 0.083 Aa	7.646 ± 0.018 BCa	7.680 ± 0.001 Da	7.677 ± 0.032 B
	MN	7.749 ± 0.038 Ab	7.948 ± 0.031 Aab	7.953 ± 0.006 Aa	7.884 ± 0.017 A

*Note:* Different uppercase letters indicate significant differences among treatments (*p* < 0.05), while different lowercase letters indicate significant differences among varied soil depths (*p* < 0.05).

**TABLE 2:** Results of a repeated‐measures ANOVA testing for differences in soil properties (SWC, pH, NH_4_^+^‐N and NO_3_^−^‐N) among N addition using soil depth as the repeated variable.

Source of variation	*df*	SWC		pH		NH_4_^+^‐N		NO_3_^—^N	
		*F*	*p*	*F*	*p*	*F*	*p*	*F*	*p*
T	3	49.35	0.000	21.47	0.000	47.83	0.000	103.27	0.000
D	2	299.04	0.000	3.66	0.041	7.88	0.002	42.98	0.000
T × D	6	13.64	0.000	3.17	0.020	4.75	0.003	16.79	0.000

Abbreviations: D, depth; T, treatment.

### 3.2 Effects of N Treatments on Soil NH_4_ ^+^‐N and NO_3_ ^−^‐N

**FIGURE 2:**
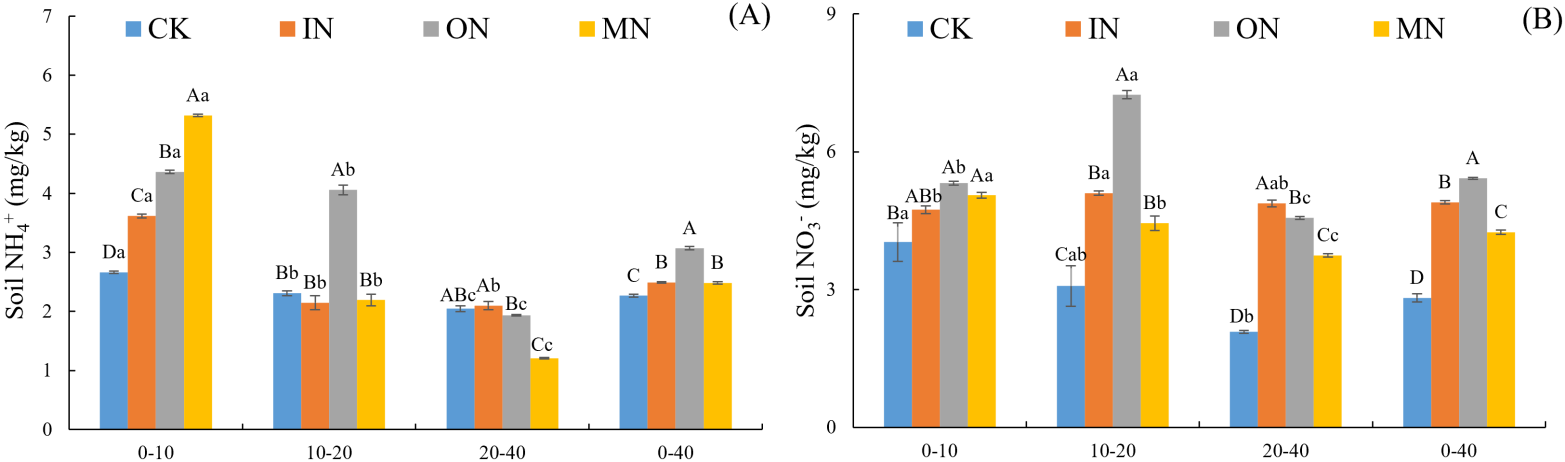
Effects of IN, ON and MN treatments on (A) NH_4_^+^‐N content and (B) NO_3_^−^‐N content of soil of saline‐alkaline grassland. IN, inorganic nitrogen; ON, organic nitrogen; MN, mixed nitrogen. Different uppercase letters in the same soil depth indicate significant differences among treatments (*p* < 0.05), while different lowercase letters indicate significant differences among varied soil depths (*p* < 0.05).

Different N treatments (IN, ON and MN) significantly increased soil NH_4_^+^‐N and NO_3_^−^‐N contents (*p* < 0.05, Figure 2). Compared to CK, soil NO_3_^−^‐N content in the 0–40 cm layer of profile increased by 73.80%, 92.44% and 50.69% under IN, ON and MN treatments, respectively, while NH_4_^+^‐N content increased by 9.9%, 35.6% and 9.5%, respectively. Soil NH_4_^+^‐N and NO_3_^−^‐N contents decreased with increasing soil depth across treatments, except in the IN and ON treatments, where the maximum NO_3_^−^‐N content was observed in the 10–20 cm layer. Repeated analysis of variance demonstrated that N addition and soil depth significantly interaction influenced the soil NH_4_^+^‐N and NO_3_^−^‐N (Table 2).

### 3.3 Soil Enzyme Activities Under Different N Treatments

**FIGURE 3:**
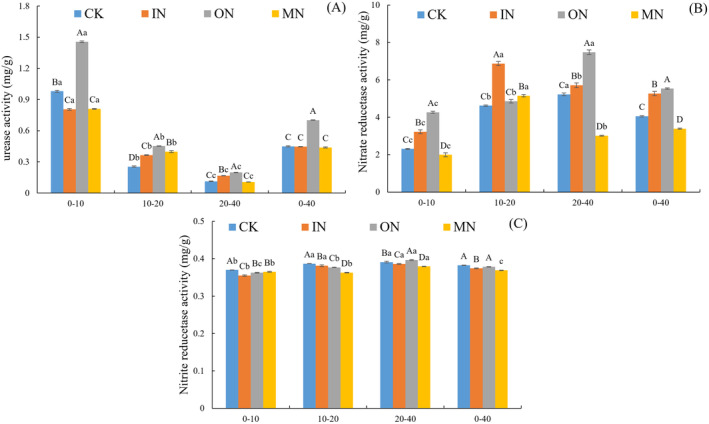
Effects of IN, ON and MN treatments on (A) urease activity, (B) nitrate reductase activity and (C) nitrite reductase activity of soil of saline‐alkaline grassland. Different uppercase letters in the same soil depth indicate significant differences among treatments (*p* < 0.05) while different lowercase letters indicate significant differences among varied soil depths (*p* < 0.05).

Significant differences were observed in soil urease and nitrate reductase activities among the treatments (IN, ON and MN) and CK (*p* < 0.05, Figure 3). In the 0–40 cm soil depth, the highest soil urease activity was found in the ON treatment (0.576 mg g^−1^), followed by IN (0.376 mg g^−1^), CK (0.365 mg g^−1^) and MN (0.356 mg g^−1^). Nitrate reductase activity was also highest in the ON treatment (6.020 mg g^−1^), followed by IN (5.377 mg g^−1^), CK (4.343 mg g^−1^) and MN (3.291 mg g^−1^). Conversely, nitrite reductase activity was highest in CK (0.385 mg g^−1^), followed by ON (0.383 mg g^−1^), IN (0.377 mg g^−1^) and MN (0.372 mg g^−1^). Urease activity decreased significantly with soil depth, while nitrite reductase activity significantly increased. Soil nitrate reductase activity increased with soil depth under CK and ON treatments, while it first increased and then decreased under IN and MN treatments. Repeated analysis of variance demonstrated that N addition and soil depth significantly interaction influenced the soil enzyme activity (Table 3).

**TABLE 3:** Results of a repeated‐measures ANOVA testing for differences in soil enzyme activity (urease) among N addition using soil depth as the repeated variable.

Source of variation	Df	Urease		Nitrate reductase		Nitrite reductase	
		*F*	*p*	*F*	*p*	*F*	*p*
T	3	1562.39	0.000	403.80	0.000	58.04	0.000
D	2	2548.11	0.000	1021.04	0.000	360.89	0.000
T × D	6	826.11	0.000	155.97	0.000	27.32	0.000

Abbreviations: D, depth; T, treatment.

### 3.4 Correlation Analysis Between Soil Environmental Factors and Enzyme Activities

Correlation analysis revealed a significant positive correlation between nitrate reductase and NH_4_^+^‐N and NO_3_^−^‐N (*p* < 0.05, Table 4) and a significant negative correlation with pH (*p* < 0.01). Nitrite reductase was significantly negatively correlated with pH (*p* < 0.01). Urease showed a positive correlation with NH_4_^+^‐N and NO_3_^−^‐N (*p* < 0.05) and a negative correlation with SWC (*p* < 0.05). There was a significant positive correlation between NH_4_^+^‐N and NO_3_^−^‐N (*p* < 0.01) and a significant negative correlation with SWC (*p* < 0.05). NH_4_^+^‐N exhibited a significant negative correlation with SWC (*p* < 0.01).

**TABLE 4:** Correlation analysis between soil properties.

	Nitrate reductase	Nitrite reductase	Urease	NO_3_^−^‐N	NH_4_^+^‐N	SWC	pH
Nitrate reductase	1						
Nitrite reductase	0.557	1					
Urease	0.753**	0.439	1				
NO_3_^−^‐N	0.611*	−0.219	0.653*	1			
NH_4_^+^‐N	0.669*	0.168	0.946**	0.826**	1		
SWC	−0.126	0.218	−0.689*	−0.602*	−0.791**	1	
pH	−0.716**	−0.861**	−0.326	0.097	−0.089	−0.431	1

** Highly significant correlation (*p* < 0.01).

* significant correlation (*p* < 0.05).

## 4 Discussion

### 4.1 Effects of Different N Treatments on Soil Properties

Increased N deposition is known to enhance terrestrial ecosystems productivity and influence soil properties significantly (Diao et al. 2022). In this study, various forms of N addition led to an increase in soil pH, contrary to the common expectation of soil acidification due to N addition (Cai et al. 2017; Guo et al. 2011). Organic N input, for example, releases ammonia via urease activity, where NO^+^ interacts with H^+^ to form NH_4_^+^, subsequently raising the soil's nitric acid levels and pH (Zhang et al. 2021; Dimkpa et al. 2020). However, in our saline‐alkaline grassland study area, increased salinity likely decreased root respiration and CO_2_ partial pressure (Wang et al. 2019), thereby promoting pH elevation and carbonate formation (Wong et al. 2010). Soil pH also rose with increasing depth, possibly due to salt leaching and accumulation in deeper layers, resulting in lower pH at the surface. Additionally, under all three N addition treatments, soil NO_3_^−^‐N content surpassed NH_4_^+^‐N content, aligning with our initial hypothesis and findings from previous studies (Vikram et al. 2023; Wang et al. 2023). High soil pH (> 6) with good aeration, NO_3_^−^‐N is the dominant inorganic N form (Keller et al. 2024), meaning that N addition promotes soil nitrification (Zheng et al. 2022), converting NH_4_^+^ to NO_3_^−^ and boosting soil NO_3_^−^‐N content.

### 4.2 Effects of Different N Treatments on Soil Enzyme Activities

Soil enzymes drive all biochemical processes within the soil, playing a crucial role in nutrient cycling and energy flow (Xu et al. 2017). In this study, organic N fertilizer significantly elevated soil urease activity (Figure 3), consistent with prior research (Ge et al. 2010) and our second hypothesis. Increase is due to organic nitrogen fertilizer raising soil organic N content, which microbes and plants then convert into inorganic forms via urease activity (Tian et al. 2022; Jian et al. 2016). This catalysis boosts soil NO_3_^−^‐N and NH_4_^+^‐N content (Figure 2). Additionally, microbes prefer organic nitrogen sources, such as glycine, because they also provide a carbon source (Zhou et al. 2023; Yang et al. 2016). The increase in soil NO_3_^−^‐N and NH_4_^+^‐N supports plant growth, leading to higher root secretions that stimulate microbial activity and further increase urease activity (Jian et al. 2016). This is supported by the significant positive correlation between soil NO_3_^−^‐N, NH_4_^+^‐N and urease activity (Table 4). Conversely, applying IN or MN fertilizer alone reduced soil urease activity compared to ON fertilizer alone, consistent with other findings (Chen et al. 2018). Organic N fertilizers, particularly in a 1:1 urea: Glycine mix, enhance soil carbon and N content, boosting microbial activity and speeding up urea and glycine decomposition (Siman et al. 2022), thereby increasing urease activity. Interestingly, there was no significant difference in urease activity between IN, MN and CK treatments, suggesting that urease activity is sensitive to IN fertilizer inhibition. Additionally, adding IN or ON significantly increased soil nitrate reductase activity, in line with our second hypothesis. Both treatments enhanced soil NO_3_^−^‐N and NH_4_^+^‐N contents (Figure 2), promoting denitrification (Xu et al. 2024; Pandey et al. 2019) and boosting nitrate reductase activity. The significant positive correlation between nitrate reductase activity and NO_3_^−^‐N and NH_4_^+^‐N contents indicates single N types enhance nitrate reductase activity. However, MN fertilizer addition significantly reduced nitrate reductase activity compared to CK (Figure 3). This was mainly because the nitrate reductase itself can convert the IN absorbed by the crop into ON (Cheng et al. 2023; Wang et al. 2018), due to MN fertilizer addition provides a higher ON content, which promoted the mineralization and decomposition of ON. This process intensifies competition between plants and microbes for effective soil N (Chen et al. 2024; Cui et al. 2022; Li et al. 2017), decreasing soil NO_3_^−^‐N and NH_4_^+^‐N and thus inhibiting nitrate reductase activity. The significant positive correlation between soil nitrate reductase and NO_3_^−^‐N and NH_4_^+^‐N in this study further confirms this (Table 4).

Different forms of N input led to lower nitrite reductase activity compared to CK, mirroring the meta‐analysis findings of Jian et al. (2016). Nitrogen addition alleviates N limitations in grassland soil but raises microbial demand for other nutrients like C and P. N input also stimulates plant growth, which increases transpiration, exacerbating soil moisture loss, further reducing nitrite reductase activity (Chang et al. 2023; Nunez et al. 2022). The weak correlation between nitrite reductase and SWC in this study supports this (Table 4). The lowest nitrite reductase activity occurred under IN and MN treatments, likely because these treatments promoted nitrite accumulation, reducing fertilizer use efficiency (Chen et al. 2004) and diminishing nitrite reductase activity. Soil pH increased with N addition, primarily because NO_3_^−^‐N levels exceeded NH_4_^+^‐N levels. Plants absorbed more NO_3_^−^‐N content, releasing OH^−^ into the soil from the plant root system (Van Beusichem, Kirkby, and Baas 1988), which slightly increased pH. Soil urease activity gradually declined with increasing soil depth, consistent with previous research (Zhong et al. 2024). On the one hand, herbaceous plant root systems are concentrated in the surface soil, where more root exudates and nutrients boost microbial activity (Lei et al. 2023; Zhang et al. 2022), leading to higher urease activity. On the other hand, surface soil also benefits from higher temperatures and better permeability, both of which promote urease activity (Dabin et al. 2019). In contrast, soil nitrate reductase and nitrite reductase activities gradually increase with soil depth, likely due to the gradual rise in soil moisture and pH (Table 1). Higher pH values (7–8.5) increase the abundance of denitrifying microorganisms (Zhou et al. 2024; Zhu, Singh, and Zhu 2019; Jetten et al. 2001), fostering nitrate and nitrite reductase production (Zhang et al. 2022) and promoting soil nitrogen cycling (Rachid et al. 2013).

### 4.3 Effects of Soil Environmental Factors on Enzyme Activities

Even small changes in the environment can significantly alter soil physicochemical properties and soil enzyme activities sensitively reflect microbial shifts in the nutrient cycling process (Yu et al. 2020). Soil enzyme activity is influenced by soil pH, temperature, moisture and nutrient content (Xu et al. 2017; Stark, Männistö, and Eskelinen 2014). This study found that soil urease and nitrate reductase activities positively correlated with NO_3_^−^‐N and NH_4_^+^‐N contents, as N is the main limiting nutrient in grasslands (Jiang et al. 2024; Harpole et al. 2016). N additions increase soil N availability, enhancing microbial decomposition of organic matter and promoting soil urease and nitrate reductase activities. However, urease activity negatively correlated with SWC, likely because the saline‐alkaline grassland conditions caused intense evaporation, reducing SWC and raising soil pH. As NO_3_^−^‐N and NH_4_^+^‐N contents approached supersaturation, they exceeded plant uptake capacity, thus promoting microbial uptake of these nutrients and increasing urease activity (Kuzyakov and Xu 2013). Additionally, the optimal pH range for soil nitrate reductase and nitrite reductase is 6.6–8.3 and either too high or too low pH will limit their activities (Šimek, Jíšová, and Hopkins 2002; Song et al. 2020), leading to a decrease in the rate of soil N cycling (Shu et al. 2023). This is further evidenced by the negative correlation between soil nitrate reductase, nitrite reductase and pH in this study (Table 4).

## 5 Conclusions

Our N addition experiment in the saline‐alkaline grasslands revealed that N treatments increased soil NO_3_^−^‐N and NH_4_^+^‐N contents, reduced SWC and slightly raised soil pH. Adding IN or ON alone significantly boosted soil urease and nitrate reductase activities, while MN treatment inhibited these enzymes. Additionally, nitrite reductase activity under IN and MN treatments was significantly lower than that under CK, with no significant changes observed between ON and CK treatments. Urease activity decreased with soil depth, whereas nitrate and nitrite reductase activities increased. These findings suggest that exogenous N, particularly ON, effectively promotes soil N cycling processes. Moreover, NO_3_^−^‐N, NH_4_^+^‐N contents and soil pH are critical in regulating enzyme activities and nutrient cycling in saline‐alkaline grassland soils. These results provide valuable insights for evaluating the mechanism underlying key enzyme activity changes in nitrogen cycling due to N deposition in saline grasslands.

## Author Contributions

**Jiangqi Wu:** conceptualization (equal), data curation (equal), investigation (equal), software (equal), supervision (equal), writing – original draft (equal), writing – review and editing (equal). **Haiyan Wang:** conceptualization (equal), data curation (equal), formal analysis (equal). **Guang Li:** conceptualization (equal), data curation (equal), funding acquisition (equal). **Fujiang Hou:** conceptualization (equal), investigation (equal), resources (equal). **Guorong Xu:** investigation (equal), resources (equal).

## Conflicts of Interest

The authors declare no conflicts of interest.

## Data Availability

The experiment datasets in the article can be accessed at Dryad Digital Repository: https://doi.org/10.5061/dryad.612jm64d7.
